# Circular RNA protein tyrosine kinase 2 aggravates pyroptosis and inflammation in septic lung tissue by promoting microRNA-766/eukaryotic initiation factor 5A axis-mediated ATP efflux

**DOI:** 10.1590/acb380323

**Published:** 2023-03-06

**Authors:** FuYan Ding, JiaLu Zhu, YanLei Hu

**Affiliations:** 1 Zhengzhou University – Central China Fuwai Hospital – Department of Adult Cardiovascular Surgical Intensive Care Unit – Zhengzhou (Henan), China.

**Keywords:** Sepsis, Lung Injury, Pyroptosis, Inflammation

## Abstract

**Purpose:**

Sepsis is characterized by an acute inflammatory response to infection, often with multiple organ failures, especially severe lung injury. This study was implemented to probe circular RNA (circRNA) protein tyrosine kinase 2 (circPTK2)-associated regulatory mechanisms in septic acute lung injury (ALI).

**Methods:**

A cecal ligation and puncture-based mouse model and an lipopolysaccharides (LPS)-based alveolar type II cell (RLE-6TN) model were generated to mimic sepsis. In the two models, inflammation- and pyroptosis-related genes were measured.

**Results:**

The degree of lung injury in mice was analyzed by hematoxylin and eosin (H&E) staining and the apoptosis was by terminal deoxynucleotidyl transferase-mediated dUTP-biotin nick end labeling staining. In addition, pyroptosis and toxicity were detected in cells. Finally, the binding relationship between circPTK2, miR-766, and eukaryotic initiation factor 5A (eIF5A) was detected. Data indicated that circPTK2 and eIF5A were up-regulated and miR-766 was down-regulated in LPS-treated RLE-6TN cells and lung tissue of septic mice. Lung injury in septic mice was ameliorated after inhibition of circPTK2.

**Conclusions:**

It was confirmed in the cell model that knockdown of circPTK2 effectively ameliorated LPS-induced ATP efflux, pyroptosis, and inflammation. Mechanistically, circPTK2 mediated eIF5A expression by competitively adsorbing miR-766. Taken together, circPTK2/miR-766/eIF5A axis ameliorates septic ALI, developing a novel therapeutic target for the disease.

## Introduction

Sepsis refers to an injury caused by the body’s own response to inflammation, severely damaging lung, kidney, liver, and cardiovascular systems^1^. Hyperinflammation-featured acute lung injury (ALI) is a serious complication of sepsis^2^. Sepsis is a major cause of infection-related deaths, affecting approximately 30 million people worldwide and causing 6 million deaths annually^3^. Apart from intensive care unit treatment, no effective treatment is currently available for severe sepsis^4^. Genomics of sepsis hint that patients present inflammation and immunosuppression with altered expression levels of most genes^5^. In addition, some molecular players have been identified to be potential targets for the treatment of organ failure in patients with sepsis, such as microRNAs (miRNAs) and circular RNAs (circRNAs)^6^. These transcripts have also been illustrated to be related to the pathogenesis of multiple organ system failures^7^. Therefore, this study explored the possible roles of circRNAs in the pathogenesis of sepsis and sepsis-induced ALI at the gene level.

CircRNAs, defined as covalently closed noncoding RNAs^8^, have multiple biological functions in a variety of cellular events, including pyroptosis and apoptosis, and are associated with sepsis^9^^,^ 10. In addition, circRNAs can regulate a variety of molecular mechanisms, including inflammation and immune responses, as well as a variety of biological processes in various metabolic organs^11^. For example, altering circ-fatty acid desaturase 2 or circ_0001679 at the gene level can inhibit sepsis-induced ALI progression^6^^,^ 12. CircRNA protein tyrosine kinase 2 (circPTK2) has attracted academic attention in the aspect of malignancy^13^^–^ 15 and has also been studied to mediate apoptosis and inflammatory response in sepsis-induced myocardial injury^16^. Notably, circPTK2 can also regulate sepsis-induced microglial activation and apoptosis in the hippocampus^17^. Since circRNAs absorb miRNA to upregulate target genes by isolating and competitively inhibiting miRNA activity^9^^,^ 12, this study intended to explore whether circPTK2 mediates septic ALI in this way.

CircPTK2-related mechanisms were studied in a mouse model of septic ALI and a model of alveolar cells stimulated by lipopolysaccharides (LPS). Furthermore, the effects of circPTK2 on lung tissue damage and inflammation were investigated, thereby providing potential insights into the clinical treatment of septic ALI.

## Methods

### Establishment of a mouse model of septic ALI

All animals underwent procedures with the approval of the Animal Ethics Review Committee of Central China Fuwai Hospital of Zhengzhou University, Heart Center of Henan Provincial People’s Hospital. Under adaptive conditions (24 ± 2 °C, 12/12 h light-dark cycle, 55–65% humidity), 60 adult male C57BL/6 mice (Hunan SJA Laboratory Animal Co., Ltd., China) were housed with enough water and food supply. Mice were randomly divided into the following four groups (n = 15/group) : Sham group, cecal ligation and puncture (CLP) group, AVV-sh-negative control (NC) group, and AVV-sh-circPTK2 group. A sepsis model was established by CLP surgery^18^. Briefly, mice were anesthetized by intraperitoneal injection of 10% chloral hydrate (10 mL/kg) and fixed on the operating table to make a midline incision (4 mm) to expose the cecum. The cecum was sutured with 3-0 silk suture at a distance of 10 mm from the tip, and punctured with a 20-gauge needle at a distance of 5 mm from the ligation. Then, the intestine was repositioned, and the peritoneum and skin were closed with sterile sutures. All mice were resuscitated by subcutaneous injection with 5 mL/100 g of saline. Sham-operated mice (n = 15) received cecal ligation but no puncture. To knock down circPTK2, shRNA adenovirus vector targeting circPTK2 (AVV-sh-circPTK2) and negative control vector (AVV-sh-NC) (20 μL, 10^7^ particles/L) were injected via tail vein one week before CLP. After 24 h of surgery, the right lower lobe of euthanized mice was resected to calculate the wet/dry (W/D) ratio^19^. Part of the lung tissue was fixed with 4% paraformaldehyde for subsequent histopathological analysis, while the remaining was frozen at –80 °C for protein or RNA extraction.

### Real-time quantitative polymerase chain reaction (RT-qPCR)

Total RNA was extracted using TRIzol reagent (Takara). To determine mRNA and circRNA expression, the iSCRIPT cDNA Synthesis Kit (Roche Life Science) was utilized to synthesize complementary DNA (cDNA) from mRNA and circRNA, while the miRNA First Strand cDNA Synthesis Kit (Sangon) was to synthesize that from miRNA. Amplification was performed on a CFX96 Touch real-time PCR instrument (Bio-Rad Laboratories) using TB Green Fast qPCR Mix (Takara). Results were analyzed using the 2^-ΔΔCt^ method. The primer sequences are shown in Table 1.

**Table 1:** Primers applied for RT-qPCR.

Genes	Primers (5’–3’)
circular RNA PTK2	Forward: 5’- ACCAGAGGAGTGGAAAATATGACA-3’
	Reverse: 5’- CATGCCAGTACCCAGGTGAG-3’
microRNA-766	Forward: 5’- AGGAGGAATTGGTGCTGGCTT-3’
	Reverse: 5’- TGGTGTCGTGGAGTCG-3’
Eukaryotic translation initiation factor 5A	Forward: 5’-CATGCCAAGGTCCATCTGGT-3’
	Reverse: 5’-AAGGTCCTCTCGTACCTCCC-3’
U6	Forward: 5’-CTCGCTTCGGCAGCACA-3’
	Reverse: 5’-AACGCTTCACGAATTTGCGT-3’
glyceraldehyde-3-phosphate dehydrogenase	Forward: 5’-CATCAACGGGAAGCCCATC-3’
	Reverse: 5’-CTCGTGGTTCACACCCAT-3’

### Hematoxylin and eosin (H&E) staining

Lung tissue was routinely dehydrated, cleared, and waxed 24 h after fixation. Next, the paraffin-embedded lung tissue was cut into 4 μm, dewaxed with xylene, dehydrated with gradient ethanol, stained with hematoxylin for 5 min and with eosin for 2 min. Finally, neutral resin was mounted and histopathological injury was evaluated under a light microscope^18^.

### Terminal deoxynucleotidyl transferase-mediated dUTP-biotin nick end labeling (TUNEL) staining

Lung sections were deparaffinized and rehydrated with ethanol at gradient concentrations of 100, 95, and 75% for 5 min. After rinsing with distilled water, proteinase K (10 μg/mL, Roche) was added for 10 min and then washed with tris-buffered saline (3 more times; 5 min each). After the addition of equilibration solution for 10 min, apoptosis was measured by a TUNEL Assay Kit (Roche, 11684795910) with 4’,6-diamidino-2-phenylindole (Roche, 216276) to stain nuclei.

### Immunohistochemistry

After routine deparaffinization and rehydration, lung tissue sections were boiled in 0.01 mol/L citrate buffer (pH 6.0) and added with 3% hydrogen peroxide to remove nonspecific background staining. Antigen-antibody complexes were developed by adding 3,30-diaminobenzidine (TL-015-HDJ, Thermo Scientific Lab Vision UltraVision ONE). Microscopic images were taken after H&E counterstaining, dehydration, and mounting.

### Enzyme-linked immunosorbent assay

Tissue and cell supernatants were examined for levels of interleukin (IL)-1β, IL-18, tumor necrosis factor (TNF)-α, and IL-6 according to the manufacturer’s protocol (Abcam).

### Cell culture

Alveolar type II cells (RLE-6TN; ATCC) were maintained in Dulbecco’s modified eagle’s medium (Hyclone, USA) containing 10% fetal bovine serum (Hyclone) and induced with 0, 1, 5, and 10 μg/mL LPS (Sigma-Aldrich) for 48 h to mimic septic ALI.

### Cell transfection

GenePharm (Shanghai, China) provided the transfection sequences including siRNA targeting circPTK2 and eukaryotic initiation factor 5A (eIF5A) (si-circPTK2, si-eIF5A), pcDNA 3.1 (pcDNA 3.1-circPTK2, pcDNA 3.1-eIF5A), miR-766 mimic/inhibitor, and corresponding negative controls. LPS-treated RLE-6TN cells (5 μg/mL) were transfected using Lipofectamine 2000 (Invitrogen) and measured by RT-qPCR after 48 h.

### Cytotoxicity assay

RLE-6TN cells plated in 96-well plates containing 2 × 10^4^ cells/well were cultured for 24 h and for another 1 h with 10 μL Cell Counting Kit-8. Analysis of absorbance was performed on a microplate reader at 450 nm.

### Flow cytometry

RLE-6TN cells were double-labeled with FAM-YVAD-FMK (pro-caspase-1) and propidium iodide (membrane pore formation) using the FAM-YVAD-FMK/PI detection kit (Cat#97, ImmunoChemistry, USA). Finally, analysis was done on a BD FACSAria flow cytometer (BD company, USA).

### ATP detection

Cell supernatants were analyzed by an enhanced ATP analysis kit (Beyotime, China)^20^.

### Western blot

Lysed samples collected by Radio-Immunoprecipitation Assay buffer (Beyotime) were measured by a bicinchoninic acid protein assay kit (Thermo Fisher Scientific). The proteins were separated using 12% sodium dodecyl sulfate-polyacrylamide gel electrophoresis and transferred to a nitrocellulose membrane which was then covered with 5% skim milk for overnight incubation with anti-pro-caspase-1 (ab179515, Abcam), gasdermin D (GSDMD; 20770-1-AP, Proteintech), plasma-membrane-bound pannexin-1 (PANX1; 12595-1-AP, Proteintech), p-p65 (3033, Cell Signaling Technology), p65 (ab86299, Abcam), eIF5A (ab32443, Abcam), and glyceraldehyde-3phosphate dehydrogenase (ABclonal, A19056, 1:1000). Next, horseradish peroxidase-conjugated secondary antibody was added for 1.5 h and enhanced chemiluminescence-developed bands were imaged using Tanon 5200 (Tanon, China) and analyzed using ImageJ software.

### Dual-luciferase reporter assay

CircPTK2 and eIF5A sequences containing putative target sites of miR-766 were synthesized and cloned intopMIR-REPORT reporter vector (Thermo Fisher Scientific), named wild-type circPTK2 (WT-circPTK2) and wild-type eIF5A (WT-eIF5A). Mutants of circPTK2 and eIF5A (MUT-circPTK2 and MUT-eIF5A) lacking the complementary site of miR-766 were also generated. After the above reporter vector was cotransfected with mimic NC and miR-766 mimic in RLE-6TN cells, luciferase activity was detected by the dual-luciferase reporter assay system (Promega) 48 h later.

### RNA immunoprecipitation (RIP) experiment

RNA-protein complexes were isolated using the Magna RIP Kit (Millipore). Cell lysates were suspended in RIP lysis buffer and detected with anti-argonaute-2 (Millipore) or mouse immunoglobulin G (Millipore). Then, protein A/G beads were supplemented and the isolated RNA was measured by RT-qPCR.

### Data analysis

The data in a normal distribution were expressed as mean ± standard deviation (SD) and compared by student t test for two groups, one-way analysis of variance for multiple groups, and Tukey’s honest significant difference for post-hoc two-group test. The data in a nonparametric distribution were represented by median and interquartile range and compared by Kruskal–Wallis H-test for multiple groups and Steel–Dwass for post-hoc two-group test. All experiments were performed in at least three biological replicates. P < 0.05 was considered statistically significant.

## Results

### Alleviating effects of circPTK2 silencing on septic ALI mice

**Figure 1:**
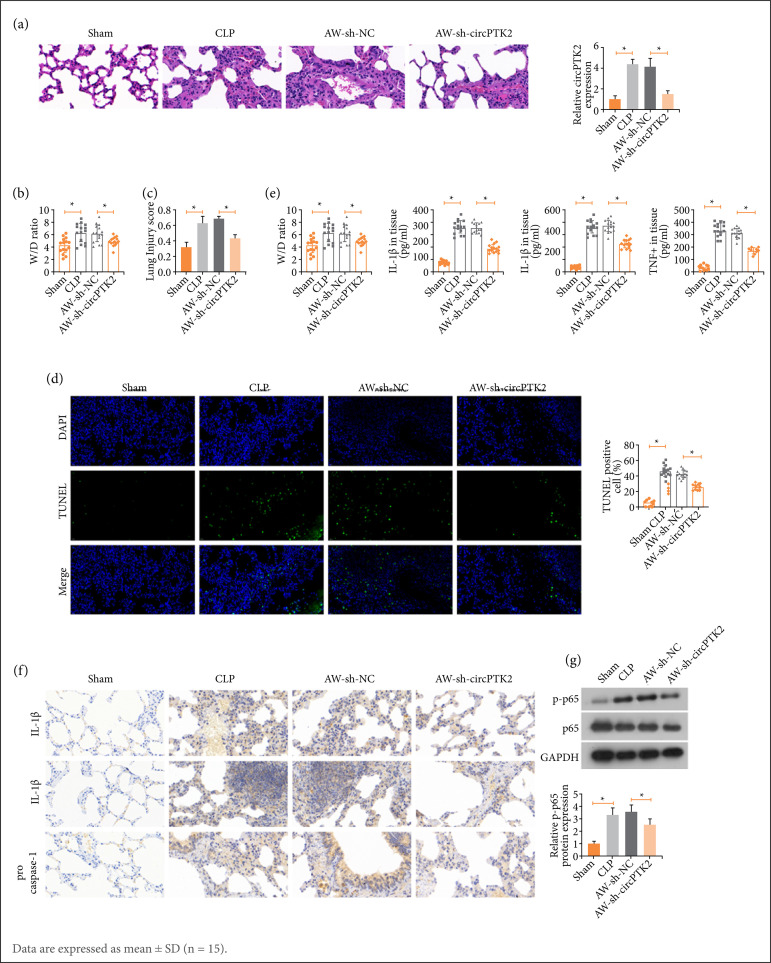
circPTK2 silencing has alleviating effects on septic ALI mice. A mouse model of septic ALI was established by CLP surgery and AVV-sh-circPTK2 was injected. **(a)** CircPTK2 expression; **(b)** W/D ratio; **(c)** H&E-stained lung tissue; **(d)** TUNEL-stained lung tissue; **(e)** Inflammatory factors in lung tissue; **(f)** Pro-caspase-1, IL-1, and IL-18 positive cells in lung tissue; **(g)** P-p65 protein expression.

A CLP-induced septic mouse model was established and injected with AVV-sh-circPTK2. CircPTK2 was increased after CLP surgery and suppressed by AVV-sh-circPTK2 (Fig. 1a). The W/D ratio of mouse lung tissue was elevated after CLP, but decreased by down-regulating circPTK2 (Fig. 1b). H&E staining depicted that the structure of lung tissue in sham-operated mice was clear without inflammatory cell infiltration. The alveolar walls of CLP-treated mice were widened, with edema and strong inflammatory cell infiltration. After knockdown of circPTK2, the inflammation and edema in the lung tissue of CLP mice were relieved, and the alveolar walls were narrowed (Fig. 1c). TUNEL staining displayed that CLP surgery promoted alveolar cell apoptosis, which could be alleviated by silencing circPTK2 (Fig. 1d). Notably, CLP surgery resulted in a significant increase in the levels of IL-1β, IL-18, IL-6 and TNF-α in lung tissue, while circPTK2 depletion had the suppressive impact on those factors. In immunohistochemistry staining, it could be seen that the CLP-induced increase in pro-caspase-1, IL-1, and IL-18 was reduced when circPTK2 was knocked down (Fig. 1f). In addition, p-p65 protein expression was increased after CLP surgery, while inhibited after knocking down circPTK2 (Fig. 1g).

### Alleviating effects of circPTK2 silencing on lung epithelial cells

**Figure 2:**
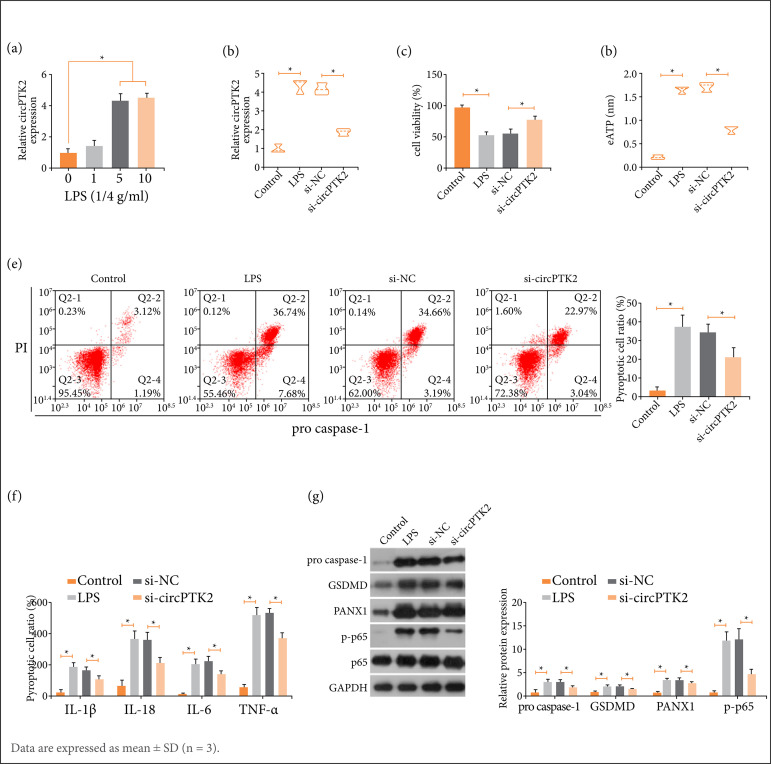
circPTK2 silencing has alleviating effects on lung epithelial cells. Alveolar type II cells were treated with LPS and transfected with siRNA targeting circPTK2. **(a)** CircPTK2 expression after LPS treatment; **(b)** After transfection, changes in circPTK2 expression; **(c)** cytotoxicity; **(d)** eATP level; **(e)** Pyroptosis; **(f)** Inflammatory factors; **(g)** Protein expression of pro-caspase-1, GSDMD, PANX1, and p-p65.

RLE-6TN under LPS conditions were transfected with siRNA targeting circPTK2. As the outcomes reflected, LPS-induced concentration-dependent increase in circPTK2 expression could be suppressed by transfection of si-circPTK2 (Fig. 2a,b). Subsequent cell analysis demonstrated the promoting effect of LPS on cytotoxicity, eATP level, pyroptosis, inflammatory response (IL-1β, IL-18, IL-6, and TNF-α), and protein levels of pro-caspase-1, GSDMD, PANX1, and p-p65. CircPTK2 deficiency alleviated LPS-mediated increases in these indicators (Fig. 2c–g).

### Sponge-like effect of circPTK2 on miR-766

**Figure 3:**
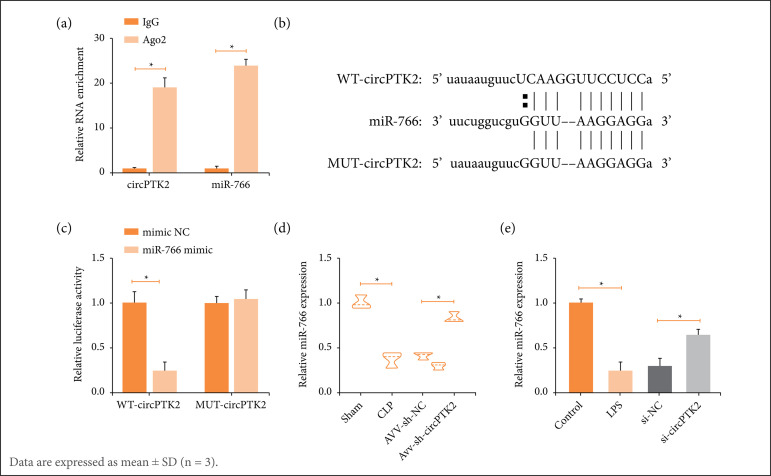
circPTK2 targets miR-766. **(a)** Enrichment of miR-766 and circPTK2; **(b)** Potential binding sites of miR-766 and circPTK2; **(c)** Targeting relationship between miR-766 and circPTK2; **(d)** miR-766 expression in CLP mice; **(e)** LPS-treated RLE-6TN cells.

Ten potential downstream miRNAs of circPTK2 were predicted through the bioinformatics website https://circinteractome.nia.nih.gov, among which miR-766 was enriched with circPTK2 in RIP experiments (Fig. 3a). Subsequently, based on the predicted binding sites (Fig. 3b), luciferase reporter vectors were constructed to ensure the targeting relationship between circPTK2 and miR-766 (Fig. 3c). Experimentally, miR-766 was down-expressed in both CLP mouse lung tissues and LPS-treated RLE-6TN cells, but was restored by circPTK2 deficiency (Fig. 3,e).

### The synergy of circPTK2 and miR-766 in lung epithelial cells under LPS conditions

**Figure 4:**
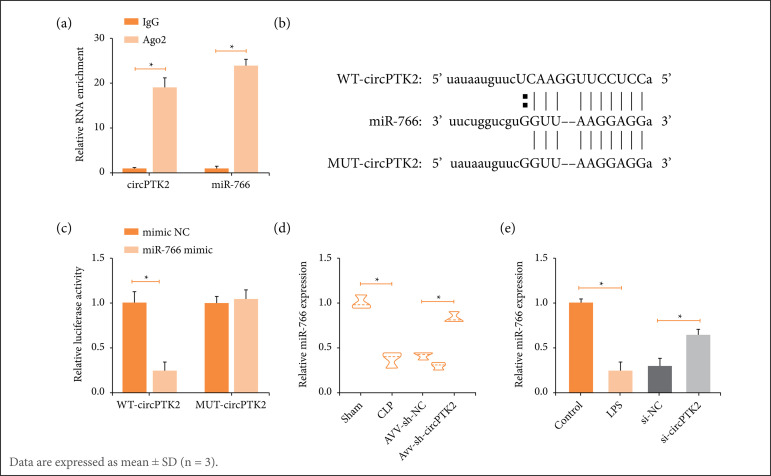
The synergy of circPTK2 and miR-766 affects lung epithelial cells under LPS conditions. pcDNA 3.1-circPTK2 and miR-766 mimic were co-transfected into LPS-treated cells. **(a)** After transfection, changes in miR-766 expression; **(b)** Cytotoxicity; **(c)** eATP level; **(d)** Pyroptosis; **(e)** Inflammatory factors; **(f)** Protein expression of pro-caspase-1, GSDMD, PANX1, and p-p65.

A cotransfection system was built with pcDNA 3.1-circPTK2 and miR-766 mimic in LPS-treated cells. pcDNA 3.1-circPTK2 up-regulated circPTK2 and down-regulated miR-766, but miR-766 mimic was the reverse of miR-766 expression (Fig. 4a). circPTK2 upregulation further increased cytotoxicity (Fig. 4b), eATP levels (Fig. 4c), pyroptosis (Fig. 4d), levels of inflammatory factors (Fig. 4e), and inflammation and pyroptosis-related proteins (Fig. 4f), and these changes were alleviated by forced expression of miR-766.

### The binding of miR-766 to eIF5A

**Figure 5:**
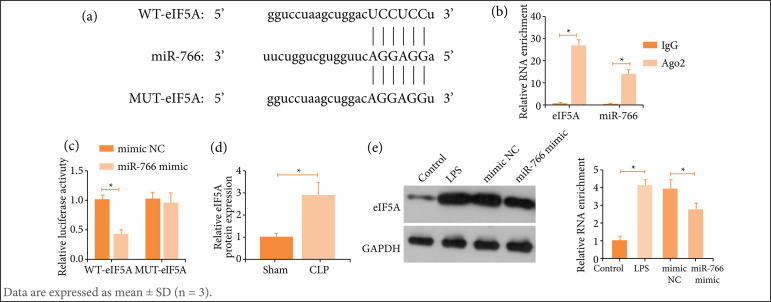
miR-766 targets eIF5A. **(a)** Enrichment of miR-766 and eIF5A; **(b)** Potential binding sites of miR-766 and eIF5A; **(c)** Targeting relationship between miR-766 and eIF5A; **(d)** eIF5A expression in CLP mice; **(e)** LPS-treated RLE-6TN cells.

Ten potential mRNAs with binding sites for miR-766 were predicted through the bioinformatics website: https://starbase.sysu.edu.cn. Significant enrichment of eIF5A and miR-766 was confirmed by RIP experiments (Fig. 5a). Dual-fluorescein reporter vectors were subsequently constructed based on the predicted binding sites and their targeting relationship was verified (Fig. 5b,c). eIF5A was abnormally high expressed in the two models of septic ALI, and was targeted by miR-766 in the cell model (Fig. 5d,e).

### CircPTK2-regulated miR-766/eIF5A axis in LPS-induced lung epithelial cell injury

**Figure 6:**
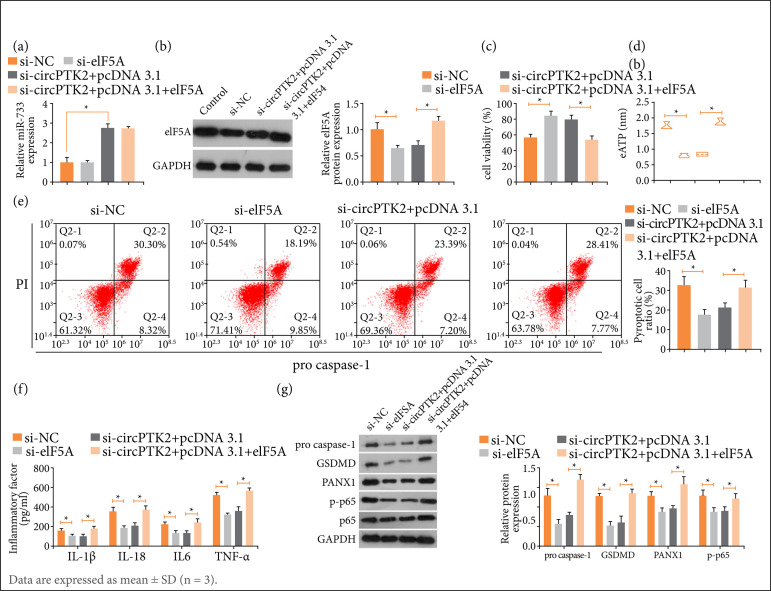
CircPTK2-regulated miR-766/eIF5A axis in LPS-induced lung epithelial cell injury. Si-eIF5A/pcDNA .1-eIF5A and si-circPTK2 were transfected into LPS-treated RLE-6TN cells. (a,b) After transfection, changes in eIF5A expression; **(c)** Cytotoxicity; **(d)** eATP level; **(e)** pyroptosis; **(f)** inflammatory factors; **(g)** Protein expression of pro-caspase-1, GSDMD, PANX1, and p-p65.

Si-eIF5A/pcDNA 3.1-eIF5A and si-circPTK2 were co-treated in LPS-treated RLE-6TN cells. Si-circPTK2-mediated eIF5A level was restored by pcDNA 3.1-eIF5A (Fig. 6a,b). Functional experiments revealed that silencing both circPTK2 and eIF5A reduced cytotoxicity, eATP levels, pyroptosis, and inflammatory response, while the effects of silencing circPTK2 were mitigated by overexpressing eIF5A (Fig. 6c–g).

## Discussion

Inflammatory mediators and cytokines are thought to initiate, enhance, and maintain sepsis-induced lung injury^21^^–^ 23. Levels of proinflammatory factors such as IL-1β, IL-18, IL-6, and TNF-α are correlated with the severity of septic ALI^24^^,^ 25. Currently, cell death is considered the driving factor in the development of ALI. It has been reported that inflammatory factors can directly promote apoptosis, further aggravating sepsis-induced lung injury^26^. In addition, pyroptosis has been thought to be involved in septic ALI^27^^–^ 29. Therefore, this study focused on exploring the effects of circPTK2 on pyroptosis and inflammation in septic ALI.

CLP is a commonly adopted *in vivo* sepsis model that can represent the human condition^30^. CLP overflows the peritoneum, leading to local infection and peritonitis, and ultimately organ failure^31^. Therefore, this study first established a CLP mouse model and evaluated the effect of circPTK2 on septic ALI, ultimately discovering that silencing circPTK2 ameliorated lung edema, pyroptosis, and inflammation in septic mice. It has been believed that the administration of LPS *in vitro* simulated septic lung injury^32^. Therefore, RLE-6TN was treated with LPS as the basis to further supplement the effect of circPTK2 on septic ALI. As the data presented, LPS treatment concentration-dependently upregulated circPTK2, and silencing circPTK2 reduced LPS-induced cytotoxicity, inflammation, and pyroptosis. eATP is now recognized as a constitutive damage-associated molecular pattern that is released by a pathogen attack^33^. The results of this study manifested that deficiency of circPTK2 attenuated LPS-induced increase of eATP levels.

CircRNAs in part inhibit the activity of miRNAs to regulate gene expression, thereby achieving functional regulation of cell biology^9^^,^ 34. This study identified a potential downstream miRNA (miR-766) of circPTK2 and found that miR-766 was down-expressed in both models of septic ALI, but circPTK2 depletion restored miR-766 levels. miR-766 has been recognized to suppress skin inflammation^35^ and attenuate ischemia/perfusion injury-induced cellular inflammation and apoptosis^36^. The paper demonstrated that miR-766 overexpression mitigated the effects of circPTK2 overexpression on cytotoxicity, eATP levels, inflammation, and pyroptosis.

eIF5A, a small protein that is highly conserved throughout evolution^37^, is closely related to pathological processes, including inflammation, proliferation, and apoptosis^38^. Notably, eIF5A down-regulation can hamper inflammation and prolong the survival of septic animals^39^. Interestingly, this study demonstrated that eIF5A was abnormally highly expressed septic ALI and that eIF5A was negatively regulated by miR-766. Furthermore, eIF5A overexpression abolished the effects of circPTK2 depletion on ATP efflux, pyroptosis, and inflammation in LPS-treated lung epithelial cells.

Although this study explains the deleterious effects of circPTK2 in sepsis, significant barriers to clinical translation remain. Due to the high mortality rate in advanced sepsis, determining whether circPTK2 can be used as a biomarker for early sepsis diagnosis would be beneficial for monitoring and warning the development of sepsis. Second, other downstream regulatory mechanisms of circPTK2 should be further explored. Furthermore, animal experiments are required to elucidate the circPTK2/miR-766/eIF5A axis-mediated mechanism in septic ALI.

## Conclusion

In short, the paper confirmed the upregulation of circPTK2 and eIF5A and downregulation of miR-766 in septic ALI models and innovatively proposed and demonstrated that circPTK2 knockdown reduces the release of inflammatory cytokines and inhibits pyroptosis through the miR-766/eIF5A axis, thereby inhibiting the progression of septic ALI. This study may provide theoretical directions for the treatment of septic ALI.

## Ethical statement

All animal experiments were complied with the ARRIVE guidelines and performed in accordance with the National Institutes of Health Guide for the Care and Use of Laboratory Animals. The experiments were approved by the Institutional Animal Care and Use Committee of Central China Fuwai Hospital of Zhengzhou University, Heart Center of Henan Provincial People’s Hospital.
